# Mass Spectrometric Detection and Characterization of Atypical Membrane-Bound Zinc-Sensitive Phosphatases Modulating GABA_A_ Receptors

**DOI:** 10.1371/journal.pone.0100612

**Published:** 2014-06-26

**Authors:** Mounia SidAhmed-Mezi, Irène Kurcewicz, Christiane Rose, Jacques Louvel, Pierre Sokoloff, René Pumain, Jacques J. Laschet

**Affiliations:** 1 Inserm, Infantile Epilepsies and Brain Plasticity U1129, Paris, France; 2 University Paris Descartes, Paris, France; 3 CEA, Gif sur Yvette, France; 4 Inserm, Centre de Psychiatrie et de Neurosciences U894, Paris, France; 5 Institut de Recherche Pierre Fabre, Neurologie & Psychiatrie, Castres, France; University of Modena and Reggio Emilia, Italy

## Abstract

**Background:**

GABA_A_ receptor (GABA_A_R) function is maintained by an endogenous phosphorylation mechanism for which the glycolytic enzyme glyceraldehyde-3-phosphate dehydrogenase (GAPDH) is the kinase. This phosphorylation is specific to the long intracellular loop I_2_ of the α1 subunit at two identified serine and threonine residues. The phosphorylation state is opposed by an unknown membrane-bound phosphatase, which inhibition favors the phosphorylated state of the receptor and contributes to the maintenance of its function. In cortical nervous tissue from epileptogenic areas in patients with drug-resistant epilepsies, both the endogenous phosphorylation and the functional state of the GABA_A_R are deficient.

**Methodology/Principal Findings:**

The aim of this study is to characterize the membrane-bound phosphatases counteracting the endogenous phosphorylation of GABA_A_R. We have developed a new analytical tool for *in vitro* detection of the phosphatase activities in cortical washed membranes by liquid chromatography coupled to mass spectrometry. The substrates are two synthetic phosphopeptides, each including one of the identified endogenous phosphorylation sites of the I2 loop of GABA_A_R α1 subunit. We have shown the presence of multiple and atypical phosphatases sensitive to zinc ions. Patch-clamp studies of the rundown of the GABA_A_R currents on acutely isolated rat pyramidal cells using the phosphatase inhibitor okadaic acid revealed a clear heterogeneity of the phosphatases counteracting the function of the GABA_A_R.

**Conclusion/Significance:**

Our results provide new insights on the regulation of GABA_A_R endogenous phosphorylation and function by several and atypical membrane-bound phosphatases specific to the α1 subunit of the receptor. By identifying specific inhibitors of these enzymes, novel development of antiepileptic drugs in patients with drug-resistant epilepsies may be proposed.

## Introduction

Neuronal inhibition is essentially mediated by GABA type A receptors (GABA_A_R) forming anionic channels [1]. They are pentameric oligomers assembled with several subunit classes that may have multiple isoforms [1]–[3]. In adult rat brain, the most abundant subunits are α1, β2, and γ2 typical for 60% to 90% of GABA_A_R [4]. The α_1_ subunit is highly expressed throughout most brain regions especially in the cortex [5]. The function of these receptors can be modulated by reversible post-translational modifications such as phosphorylation-dephosphorylation [6]–[8]. Each subunit has four transmembrane domains and a large intracellular loop (I_2_) between transmembrane domains 3 and 4 [2], [9], containing consensus phosphorylation sites for both Ser/Thr and Tyr protein kinases [10]–[12]. The related modifications by several kinases have multiple effects such as direct modulation of the channel function [13], or receptor trafficking between synaptic sites and intracellular compartments [14], [15]. In addition dephosphorylations by protein phosphatases (PP) have been shown to reverse the action of these kinases. For instance, PP1 and PP2B phosphatases dephosphorylate the β1–3 and γ2 subunits [16], [17], whereas PP2A dephosphorylates β3 subunits [18], [19].

Recently, a new concept of GABAergic inhibition modulation by glycolysis has been described. The glyceraldehyde-3-phosphate dehydrogenase (GAPDH) a key glycolytic enzyme has been identified as a kinase for the GABA_A_R α1 subunits [20]. This endogenous kinase is directly tied to the receptor at the neuronal membrane and phosphorylates the α1 subunits at two identified serine and threonine residues. This phosphorylation of the α1 GABA_A_R subunit has been termed “endogenous” since it does not require any exogenous kinase or kinase activator [21].

We have previously shown that this phosphorylation prevents rundown of the GABAergic responses on acutely dissociated pyramidal neurons from rat cortex. In addition, an unknown membrane-bound phosphatase dephosphorylates the GABA_A_R α1 subunit and opposes the endogenous phosphorylation [22]. Protein phosphatases modulate the responsiveness of individual synapses to neural activity [16]. Ser/Thr protein phosphatases are expressed in many cell types and cellular compartments, and are regulated via several mechanisms [23]. They are classified into phosphoprotein phosphatases (PPP's) and metal-dependent protein phosphatases (PPM's), families defined by distinct amino acid sequences and 3-D structures.

Alterations in GABA_A_R expression and function lead to various neurological diseases including epilepsy, anxiety and schizophrenia [24]. Studies carried out on human brain tissue of patients with drug-resistant epilepsies show that both endogenous phosphorylation and function of GABA_A_R are deficient in the cortical epileptogenic zones [25]. Enhancing the GABA_A_R endogenous phosphorylation state by inhibiting unknown membrane-bound phosphatase(s) would maintain GABA_A_ receptor function and therefore prevent seizures to occur. We propose this modulatory mechanism as a new target for the development of antiepileptic molecules active in drug-resistant epilepsies.

We have developed an electrophoresis autoradiographic method to measure the GABA_A_R endogenous phosphorylation/dephosphorylation in washed cortical membranes [22]. However, this technique is not suitable to characterize the unidentified membrane phosphatase. The first goal of the present study was to elaborate an alternative approach to detect and characterize the membrane-bound phosphatases. Here, we describe a rapid and highly sensitive method for the quantification of phosphatase activity in washed brain cortical membranes. Further, we aimed at characterizing the kinetics and the pharmacological profile of these enzymes using the novel methodological tool proposed here. We show that the pharmacological profiles of these phosphatases are atypical and do not correspond to classical phosphatases. Our biochemical and functional results bring new insights on these membrane-bound phosphatases specific to GABA_A_R α1 subunit.

## Materials and Methods

### Synthetic peptides design

**Figure 1:**
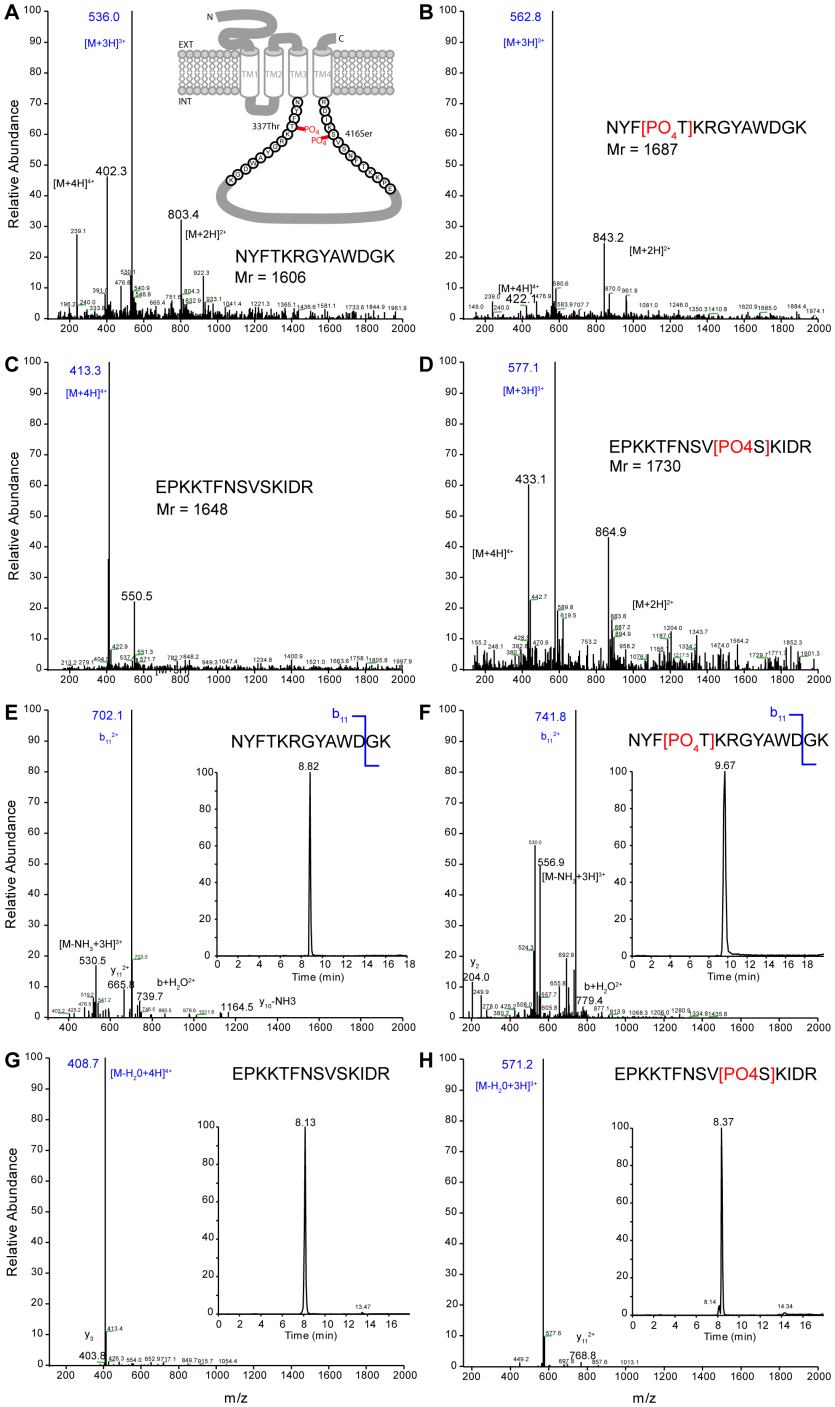
Mass spectrometry (MS) and liquid chromatography (LC)-MS/MS analysis of N- and C-terminal peptides. The insert (A) shows the representation of the α1 subunit TM3-TM4 intracellular loop (I_2_α1) of type A gamma-amino butyric acid receptor (GABA_A_R). The sequences indicate the synthetic N- and C-terminal peptides (AA334–346 and AA407–420 respectively) used in this study, with the two identified phosphorylation sites (PO_4_) on threonine (337Thr) and serine (416Ser) residues involved in the endogenous phosphorylation. EXT, extracellular; INT, intracellular. These peptides are detected in positive mode electrospray ionization (ESI) from m/z 200 to 2000 (mass in Da to charge ratio). The most abundant peptides (in blue) are selected for the subsequent ESI-MS/MS studies (parent peptides) and the other labeled ions have two, three or four charges. MS of native (A), and phosphorylated (B) N-terminal peptides. MS of native (C) and phosphorylated (D) C-terminal peptides. The major fragment ions produced by collision induced dissociation of parent peptide ions are labeled and the nature of fragmentation is indicated by “b” (N-flanking) or “y” (C-flanking) when the ion is broken at peptide bounds (break position in the amino acid sequence) or by losses (−) or gains (+) of small neutral molecules (water, ammonia). The highest intensity peaks used for identification and quantification of the different peptides are indicated in blue. LC-MS/MS analysis was performed simultaneously for the native and phosphorylated peptides. (E) Spectrum of native (F) phosphorylated N-terminal peptides. (G) Spectrum of native (H) phosphorylated C-terminal peptides. The inserts show the chromatograms of the chosen fragmentation products.

Four peptides were synthesized by Sigma-Genosys the amino acid sequences of which include the two identified endogenous phosphorylation sites of the I_2_ loop of GABA_A_R α1 subunit (sequences are identical in the human, rat, bovine and mouse species). The minimal consensus [NXX(T/S)K] of GAPDH-related phosphorylation site is present in several GABA_A_R alpha subunits (α1, α2, α3, α5), the I_2_ loop sequences being different in each subunit. We were however able to design synthetic phosphopeptides specific for the α1 subunit as substrates. Indeed, making a BLAST with the designed peptide sequences (N- and C-terminal) against mammalian EMBL-EBI database entries showed sequence identity only for the GABA_A_R α1 subunit. The loop N-terminal (TM3 domain flanking) native peptide NYFTKRGYAWDGK and its threonine-phosphorylated derivative NYF[pThr]KRGYAWDGK corresponds to the 13 amino acids 334–346 of the α1 subunit, whereas the loop C-terminal (TM4 domain flanking) native peptide EPKKTFNSVSKIDR and the serine-phosphorylated derivative EPKKTFNSV[pSer]KIDR correspond to the 14 amino acids 407–420 of the same subunit (**Figure 1**). These peptides contain the minimum consensus sequence for phosphorylation by GAPDH, and are used as substrates to characterize the membrane-bound phosphatases of the GABA_A_R α1 subunit.

### Washed cortical membrane preparation

Washed membranes were prepared mainly from bovine and rat brain cortex. The human cortex sample was frozen on dry ice in the operating room and then thawed for assay. The patient was a girl aged 16 years at the time of curative surgery. She was suffering from epilepsy associated to a non-malignant ganglioglioma. The tissue sample was taken outside the tumor in the posterior part of the right inferior temporal gyrus, with information and consent of the patient and of her parents.

The gray matter was homogenized in a Polytron system in 10 volumes of an ice-cold buffer containing 50 mM Tris pH 7.4, 0.32 M sucrose, 5 mM EDTA and 1 mM EGTA. The homogenate was centrifuged at 800–100 *g* for 10 min at 4°C. The supernatant (S1) was collected and centrifuged at 100 000 *g* for 30 min at 4°C. The pellet (P2) was osmotically shocked in 10–20 volumes of ice-cold water and sonicated. The microsome suspension was centrifuged at 100 000 *g* for 30 min at 4°C. The pellet (P3) corresponding to washed membranes was suspended in a buffer containing 10 mM Hepes (pH 7.4) and 10 mM KCl, and then centrifuged again. The last pellet (P4) was stored at −80°C.

Total protein concentrations were determined using the Bradford-based protein assay reagent (Bio-Rad) with Coomassie Brilliant Blue G-250. Calibration was done with a 20–500 µg/ml BSA range. Absorbance was measured at 570 nm.

### Liquid chromatography-electrospray ionization mass spectrometry

#### Instrumentations

The HPLC-mass spectrometric analysis was performed using a LCQ Advantage ion-trap mass spectrometer (ThermoFinningan) equipped with electrospray ionization (ESI) source in the positive mode. The ESI source was coupled online with liquid chromatography (nano- or micro-LC) systems.

#### Nano-HPLC chromatography

Eluted peptides were separated with Ultimate 3000 system (Dionex). The peptide preparations were loaded under different conditions through an autosampler (Dionex) onto a reverse-phase C18 capillary column (packed C18 PepMap 15 cm *L*×180 µm ID×3 µm *d*p, 100 Å-Dionex). Separation was achieved with a gradient elution program. The sample injection-loop was of 2 µl when the microcapillary nano-LC system was used. The peptide mixture (native and phosphorylated peptides) was eluted from the column with gradients in the mobile phase from *A* (5% acetonitrile/0.1% formic acid/94.9% water, v/v/v) to *B* (80% acetonitrile/0.1% formic acid/19.9% water, v/v/v). LC separation was resolved at the flow rate of 4 µl/min, by the following gradient conditions: 0–4 min 0–20% *B*, 4–14 min 20–30% *B*, 14–15 min 30–100% *B*, 15–17 min 100% *B*, 17–20 100–0% *B*. At the end of the run, the column was equilibrated in solvent *A* for 3 min. The elution solvents were filtered through a membrane filter (type HA 0.45 µm, Millipore).

#### Micro-HPLC chromatography

Chromatographic separations in micro-flow were conducted on a reversed phase C18 capillary column (150 mm *L*×2.1 mm ID×5 µm *d*p, 100 Å- Atlantis, Waters) at a flow of 250 µl/min. Injection loop was of 10 µl. Solvent and gradient are the same as in nano-LC. The chromatographic system is Surveyor MSPump (ThermoFinningan) coupled to the autosampler injector Surveyor (ThermoFinningan).

After separation the eluted peptides were directly electrosprayed into the LCQ mass spectrometer at a voltage of 5 kV. The capillary voltage was 3 V, and the temperature was kept at 280°C. The sheath (helium) and auxiliary gas (nitrogen) flow-rate was set at 35 and 15 (arbitrary units) respectively. Helium was used as a collision gas for fragmentation of ions. A full-scan mass spectrum (m/z 200–2000) was followed by fragmentation by CID (collision-induced dissociation) of the most abundant peak from the full-scan mass spectrum (parent ion), using 25% of the normalized collision energy for obtaining MS/MS spectra. The absolute signal intensity of products ions was used to display the chromatogram allowing the quantification of samples and standards by integrating area of the specified peaks.

#### Optimization of LC-MS and LC-MS/MS conditions

The LC-MS conditions were optimized by monitoring chemical parameters of standard N- and C-terminal peptides dissolved in the medium used afterwards for phosphatase activity assays. The buffer medium contained Hepes only (10 to 50 mM; pH 7.3) since Tris decreased signal sensitivity. The Mg^2+^ ions up to 100 mM did not produce any significant signal decrease. The enzymatic reaction was stopped with ice-cold 10% acetic acid instead of 0.1 M HCl, removing the signals. These conditions enabled us to obtain adequate media for assaying phosphatase activities through measurements of the two native and phosphorylated peptide forms. For the LC-MS/MS method, the collision gas (He) pressure and collision gas voltage were adjusted to get the highest signal of the ionized product for each peptide.

#### Data analysis

Chromatographic data acquisition, peak integration and quantification were performed using the *Xcalibur LC-Quan* software package. Mass analysis to determine the nature of fragments in MS/MS was done on the online EXPASY proteomic server using the ‘ProteinProspector’ tool for MS products (http://www.expasy.ch).

### Phosphatase assays using phosphopeptides

Phosphatasic activity of bovine, rat and human washed cortical membranes was measured by using synthetic phosphopeptides as substrates. Reactions were performed in presence of 50 µg/ml (total proteins) of washed cortical membrane, 10 mM Hepes-Tris pH 7.4 and 1 mM MgCl_2_. Enzymatic reactions were initiated by adding 10 µM of phosphopeptide incubated for 10 min at 30°C and stopped by adding ice-cold acetic acid at a final concentration of 10% (w/v). Following a 800 *g* centrifugation during 10 min at 4°C, the supernatants were analyzed by LC-MS/MS. This centrifugation step was critical to remove membrane suspension that interferes with nano-LC and MS analysis. For these reasons, and to avoid micro-capillary column obstruction we have preferentially used the micro-LC column to characterize the pharmacological profile of phosphatasic activities.

Quantification of appearance of the native peptide and disappearance of the phosphopeptide in different conditions was performed using the calibration curve plotted using the Xcalibur software (P/N 64018-XCALI version 1.3). The calibration curve and linear regression were constructed using a series of standards, native and phosphorylated equimolar mixture, N- or C-terminal peptides at different concentrations 0.5, 1, 2, 5 and 10 µM (n = 2) in 10 mM Hepes pH 7.4, 1 mM Mg^2+^ and 10% acetic acid, which was the same milieu condition as in the phosphatase assay after stopping reactions. The linear regression represents peak area of MS/MS chromatogram spectra depending on the peptide concentration (**Figure S1)**.

To analyze the impact of washed cortical membranes and incubation process on the native and phosphorylated peptides, calibration standards at different concentrations were prepared in the biological matrix using washed cortical membranes (50 µg/ml of total proteins) in 10 mM Hepes, 1 mM Mg^2+^ and acetic acid 10% (w/v) incubated at 30°C for 10 min. For phosphopeptides, acetic acid was added before washed cortical membranes in order to prevent the dephosphorylation mechanism (**Figure S2)**. The total amount of the [native + phosphorylated] peptides retrieved after reaction incubations and LC-MS/MS analyses was constant and identical to the phosphopeptide amount added at the starting time (**Figure S3)**.

### Enzyme kinetics

The best conditions of incubation time and total protein concentration of washed cortical membranes were determined for assaying phosphatase activity with 10 µM of pI_2_α1 phosphopeptide. Protein concentration varied from 1 to 300 µg/ml and incubation time from 0.25 to 30 min, at fixed temperature of 30°C as in previous studies [22]. Initial velocity of the enzymatic reaction was determined by linear regression (slope value). The optimal conditions were obtained with 50 µg/ml total membrane protein concentration and 10 min incubation time. The effects of the substrate effects were measured by varying the pI_2_α1N-P and pI_2_α1C-P concentrations between 3 and 75 µM. Saturation plots were generated via non-linear fit to the *Michaelis-Menten* equation. The affinity constant (*K_m_*) and maximum velocity (*V_max_*) were evaluated from this curve fitting using the *Prism 5* computer program (GraphPad). Double-reciprocal plots with the *Lineweaver-Burk* equation were also drawn by linear fits using the same software.

### Pharmacological profiling of phosphatase activities

Different effectors were tested by addition to the incubation medium at variable concentration: the dicationic ions Mg^2+^, Ca^2+^, Zn^2+^ were used as chloride, the polycation spermine as hydrochloride, the anion F^−^ as sodium fluoride (NaF), and the inhibitors of some known phosphatases okadaic acid and the two (+/−) enantiomers of *p*–bromotetramisole oxalate (*p*-Br-t). For the inhibitory effects, relative 50% inhibition concentration (IC_50_) values were either determined graphically with a log-scale for the concentration axis or by the ‘log (inhibitor) vs. response’ nonlinear fit of *Prism* 5 software (GraphPad), considering activity in the absence of inhibitor as 100%. However, when Zn^2+^ was tested with the N and C-terminal peptides as substrates, a step was observed that was considered as an intermediate reference value to determine two sequential IC_50_.

### Electrophysiological approach

Adult Sprague Dawley rats (35–40 days of age) were anesthetized by isoflurane and killed by decapitation. The brains were removed quickly and placed in cold artificial cerebro-spinal fluid (Ringer) containing (in mM) 126 NaCl, 3 KCl, 26 NaHCO_3_, 1.25 Na_2_HPO_4_, 2.5 CaCl_2_, 1.5 MgCl_2_, 10 D-glucose, saturated with carbogen gas (O_2_:CO_2_, 95:5%) pH 7.4. Cortical neurons were acutely dissociated from 400 µm thick slices by incubation in 3 mg/ml protease-XXIII (Sigma) at 32°C, followed by mechanical dissociation. After washing, the cells were transferred in a solution containing (in mM) 135 NaCl, 3 KCl, 2 CaCl_2_, 10 Hepes, 1 MgCl_2_, 7 TEA-Cl, 10 D-glucose, and 1 µM TTX, pH 7.4. Pyramidal neurons were recorded using borosilicate glass pipettes (4–5 MΩ) filled with a solution containing (in mM) 130 CsF, 10 CsCl, 4 NaCl, 0.5 CaCl_2_, 10 Hepes, 5 EGTA, and 7 Mg-ATP. Okadaic acid at 10 µM was added to this intracellular milieu for specific experiments.

Whole-cell peak currents induced by GABA (100 µM; fast applications of 1 sec pulses every 3 min) were measured for each application and normalized to the maximal response. This maximal response was observed within 0–6 min following the seal of the patch since there was an initial run-up in some cases. The holding potential was −80 mV such that GABA evoked inward currents (Cl^−^ equilibrium potentials were measured close to −40 mV).

### Chemicals reagents

Zinc chloride, (−)-*p-*bromotetramisole oxalate and sodium fluoride were purchased from Fluka Chemika. MgCl_2_ was obtained from Prolabo. HPLC grade water *plus* and acetonitrile were purchased from Carlo-ERBA Reagents. Formic Acid was purchased from ACS-For analysis. Okadaic acid, (+)-*p-*bromotetramisole oxalate, spermine, albumin from bovine serum (BSA), protease-XXIII, NaCl, CsCl_2_, CsF, CaCl_2_, KCl, Hepes, D-glucose, EDTA, EDTA, sucrose, tetrodotoxin (TTX), tetraethylammonium choride (TEA), γ-aminobutyric acid (GABA), protease inhibitor cocktails were purchased from Sigma-Aldrich. Deionized-distilled water was used for preparing buffers and solutions, except for LC-MS.

### Statistical analysis

Data points are expressed as mean ± standard error to the mean (S.E.M.) of repeated measures (n). Non-linear regressions for the kinetics parameters (and corresponding errors), and statistical comparison between two linear regressions by the ‘slope test’ (case of N-terminal substrate) were assessed using the *Prism* 6 software (GraphPad). For cluster analysis, ANOVA and *post-hoc* tests, the *JMP* 10 software (SAS Institute Inc) was used. In all cases the alpha error was set at 5%.

### Ethics statement

All animal experimental procedures were performed in accordance with the European Communities Council Directive (86/609/EEC) and were approved by the Animal Experimentation Committee of Paris Descartes University.

The sample of human brain cortex was taken from a registered collection called “NeurochirEpilepsie” (DC-2011-1378) affiliated with the biobank CRB-NSPN of Sainte Anne's Hospital Center (CHSA), with approval of the local ethical committee (CPP Ile-de-France II, September 12^th^ 2011).

## Results

The first characterization of the GABA_A_R α1 subunits dephosphorylation were performed using ^33^P-autoradiographic measurements on gel electrophoresis [22], a reliable method but too laborious for routine assays. An efficient method was therefore lacking for rapid assays of the membrane-bound phosphatase activity counteracting the endogenous phosphorylation by GAPDH. We therefore developed a novel tool.

### Analytical tool for phosphatase assays specific to GABA_A_R α1 subunit using phosphopeptides and LC-MS/MS

#### Designing synthetic peptides for the α1 subunit of GABA_A_R

Two fragments of the mammalian GABA_A_R intracellular loop I_2_ of the α1 subunit (I_2_α1; 87 AA-length) including the two phosphorylated sites at threonine and serine residues were synthesized to study the corresponding phosphatases. The N-terminal peptide corresponds to the initial sequence of the I_2_α1 intracellular loop precisely adjacent to the TM3 domain, whereas the C-terminal peptide corresponds to the end-sequence of the I_2_α1 flanking precisely the TM4 domain (**Figure 1A** **insert**). For each fragment, a native and a phosphorylated form were synthesized by Sigma-Genosys, with distinct molecular masses. We used these peptides as useful probes to investigate experimental conditions for the retention and separation of the native and phosphorylated forms by reversed-phase liquid chromatography (LC) and electrospray ionization (ESI) for a full scan mass spectrum.

#### Full-MS detection and characterization of peptides

Sequential mass spectra for simultaneous analyses of N- and C-terminal peptides were obtained from LC-MS using a positive mode full mass scan (full-MS; m/z 200–2000). The native and phosphorylated N-terminal peptides showed that the main ions in the MS spectrum were multiple-protonated molecular ions (M+nH^n+^).

Native (pI_2_α1N, *Mr* = 1606 Da) and phosphorylated (pI_2_α1N-P, *Mr* = 1687 Da) N-terminal peptides show respectively dominant triple-charged ions [M+3H]^3+^ at m/z 536.0, and 562.8 m/z (**Figure 1A, B**). Full-MS of C-terminal peptides pI_2_α1C and pI_2_α1C-P with the molecular mass *Mr* = 1648 Da and *Mr* = 1730 Da respectively, gave rise to dominant ions at m/z 413.3, a quadruple-charged molecular ions [M+4H]^4+^ (**Figure 1C**) and at m/z 577.1, a triple-charged molecular ions [M+3H]^3+^ (**Figure 1D**). We chose these ions for detection of the peptides in tandem MS (MS/MS).

#### LC-MS/MS analysis of N- and C-terminal peptides

The parent ions were selected and fragmented for precise peptide identifications. The MS/MS spectrum of N-terminal native and phosphorylated peptides dominant double-charged ions were detected at respectively m/z 702.1 and 741.8, at LC retention times of respectively 8.82 min and 9.67 min (**Figure 1E, F** **and inserts**). These ions corresponded to the b_11_ fragment ions (b_11_^2+^) for the native and phosphorylated peptides. The MS/MS products for the native C-terminal peptide showed at m/z 408.7 a dominant fragment ion [M-H_2_O+4H]^4+^ with four charges and with a loss of one H_2_O molecule, while the fragmentation of the phosphopeptide presented at m/z 571.2 a dominant triple-charged ion [M-H_2_O+3H]^3+^ with a loss of one H_2_O molecule (**Figure 1G, H**). These peptides were eluted at 8.13 min for the native peptide and at 8.37 min for the phosphopeptide (**Figure 1G, H** **inserts**). These methods allowed rapid, sensitive and specific simultaneous determinations of the native and phosphorylated forms, qualitatively and quantitatively.

### Phosphatasic activity assays using the LC-MS/MS method and the phosphopeptides as substrates

**Figure 2:**
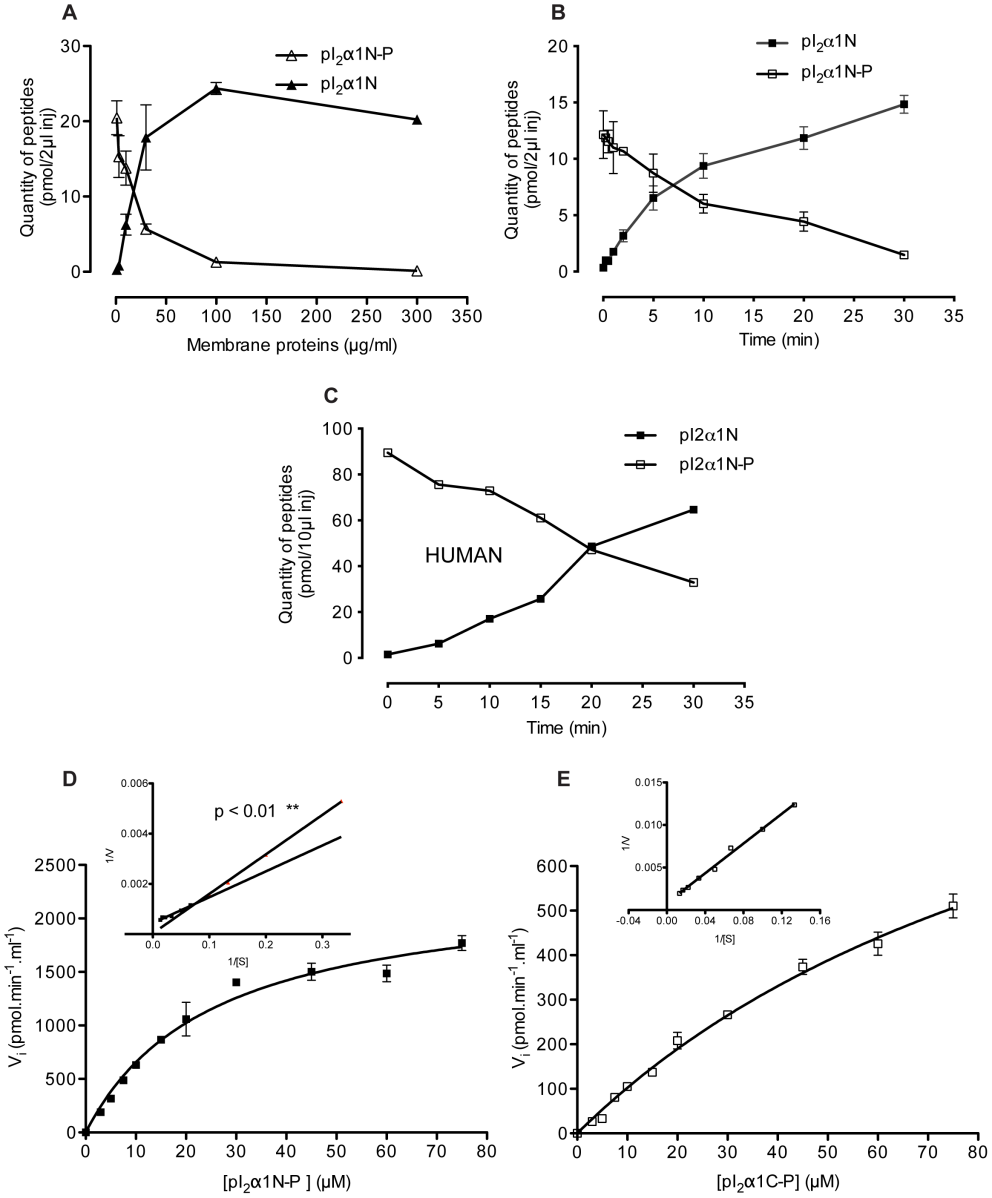
LC-MS/MS detection and kinetics analysis of GABA_A_R α1-subunit phosphatase activities. (A) Dose-dependency at various protein concentrations; (B) time course of phosphatase activities using 50 µg/ml of total proteins of washed cortical membranes from bovine brain and (C) from human epileptic tissue. The N-terminal phosphopeptide (pI_2_α1N-P) used as substrate at 10 µM was incubated during 10 min at 30°C in 10 mM Hepes (pH 7.4) in the presence of 1 mM Mg^2+^. The enzymatic reactions were stopped with 10% acetic acid and the samples were analyzed by LC-MS/MS as in figure 1E, F. The dephosphorylation rate was quantified by measuring simultaneously the quantity of the produced native peptide (pI_2_α1N) and of the remaining phosphorylated substrate (pI_2_α1N-P). Error bars represent SEM of two experiments. Kinetic analysis of GABA_A_R α1-subunit phosphatase activities was investigated using N-terminal (pI_2_α1N-P) and C-terminal (pI_2_α1C-P) phosphopeptides. All experiments were carried out with 50 µg/ml membrane proteins concentration, incubated at 30°C during 10 min, in presence of different concentrations of substrates. (D) Saturation plot of the initial velocity V_i_ versus [pI_2_α1N-P] and Lineweaver-Burk plots of 1/V versus 1/[pI_2_α1N-P] (inset) showing two significantly different slopes. (E) Saturation plot of V_i_ versus [pI_2_α1C-P] and Lineweaver-Burk plot of 1/V versus 1/[pI_2_α1C-P] (inset). The data points are means ± SEM of 3 experiments.

We used initially as substrate for phosphatase assays the N-terminal phosphopeptide PI_2_α1N-P incubated with washed cortical membranes of bovine brain cortex as the enzyme source (**Figure 2A, B**). In one pilot test we used human brain cortex obtained during neurosurgery to assess that this method can be applied to human frozen tissue (**Figure 2C**). A specific phosphatase activity was detected in all assays. **Figure 2A, B, C** shows the disappearance of phosphopeptide (PI_2_α1N-P) versus enzymatic production of native peptide (PI_2_α1-N) by varying concentration of total membrane proteins and incubation time. It shows the presence of an important membrane-bound phosphatase activity. The corresponding enzymes(s) recognized and dephosphorylated efficiently the α1-subunit substrates, and dephosphorylated the phosphopeptide. The enzyme activity was linear up to 50 µg/ml (total proteins) as shown in **Figure 2A**. The time course curves showed essentially a linear response up to 10 min (**Figure 2B**). These parameters (concentration of enzyme and time incubation) were further applied to all the enzymatic assays and related pharmacological studies, using both N- and C-terminal phosphopeptides as substrates.

### Enzyme kinetics of membrane-bound phosphatase activities

The phosphatase kinetics parameters were determined with either PI_2_α1N-P or PI_2_α1C-P as synthetic substrates (**Figure 2D, E**). The results were obtained by fitting data into the *Michaelis-Menten* equation. The phosphatase activities showed an eightfold reduction in catalytic efficiency (V_max_/K_m_) for the phosphoserine peptide (PI_2_α1C-P) compared the phosphothreonine peptide (PI_2_α1N-P), due to a combined K_m_ increase and V_max_ decrease (**Table 1**).

**Table 1:** Kinetics parameters of the phosphatase activities dephosphorylating the N- and C-terminal phosphopeptides.

*Substrate Name*	*Peptide sequence*	*V_max_* (pM.min^−1^.ml^−1^)	*K_m_*(µM)	*Efficiency V_max_. K_m_^−1^* (pM.min^−1^.ml^−1^. µM^−1^)
**pI_2_α1N-P**	NYF**[PO_4_T]**KRGYAWDGK	2320±130	25.4±3.3	91.4
**pI_2_α1C-P**	EPKKTFN**[PO_4_S]**VSKIDR	1290±150	116±20	11.1

Experimental conditions are detailed in Materials and Methods. Values are means ± SEM of two or three experiments.

The phosphatase activities have two apparent affinities for the N-terminal phosphopeptide as indicated by the *Lineweaver-Burk* double-reciprocal plot (**Figure 2D** **, insert**). The two regression lines of the plot were significantly different as assessed by the slope test (p<0.01**). This apparent heterogeneity suggests the presence of at least two different species of membrane-bound phosphatases. The phosphatase activities are more efficient at the phosphothreonine peptide. At least one phosphatase species can recognize phosphoserine as a substrate (single apparent slope) (**Figure 2E** **, insert**). This feature can be used to discriminate some of the membrane-bound phosphatases.

### Pharmacological profiles of membrane-bound phosphatases

The phosphatase activities were inhibited by the ions Zn^2+^, Ca^2+^ and F^−^, Zn^2+^ showing the strongest inhibitory effect. Sodium fluoride, a common inhibitor of PP1, PP2A, and PP2B but not of PP2Cα and -β [26] inhibited phosphatase activities with an IC_50_<2 mM. Okadaic acid, a potent phosphatase inhibitor of PP1, PP2A and PP2B with distinct IC_50_, also inhibited membrane-bound phosphatases.

#### Metal ion dependence of dephosphorylation

We had previously demonstrated that endogenous phosphorylation requires Mg^2+^ ions in the incubation milieu, and that in the presence of these ions GABA_A_R dephosphorylation occurs spontaneously [22]. Indeed the activities of the PPM family require divalent cations such as Mg^2+^ and Mn^2+^, Mg^2+^ showing a stronger effect [27]. Other ions such as Ca^2+^ and Zn^2+^ competitively inhibit the phosphatases by blocking the Mg^2+^/Mn^2+^ binding site [28].

Therefore, assays were performed to determine which divalent cations are effective in modulating the phosphatase activities using the N-terminal phosphopeptide as substrate.

#### Mg^2+^ ions

Increasing Mg^2+^ concentrations from 1 µM to 1 mM induced a moderate but not linear increase of the phosphatase activities (data not shown). A substantial phosphatase activity was still present without any addition of Mg^2+^, even when the chelators EDTA (5 mM) and EGTA (1 mM) were added (data not shown), indicating a heterogeneity of the dependence of the membrane-bound phosphatases to this ion. The other cations were tested in presence of 1 mM Mg^2+^.

#### Zn^2+^ ions

**Figure 3:**
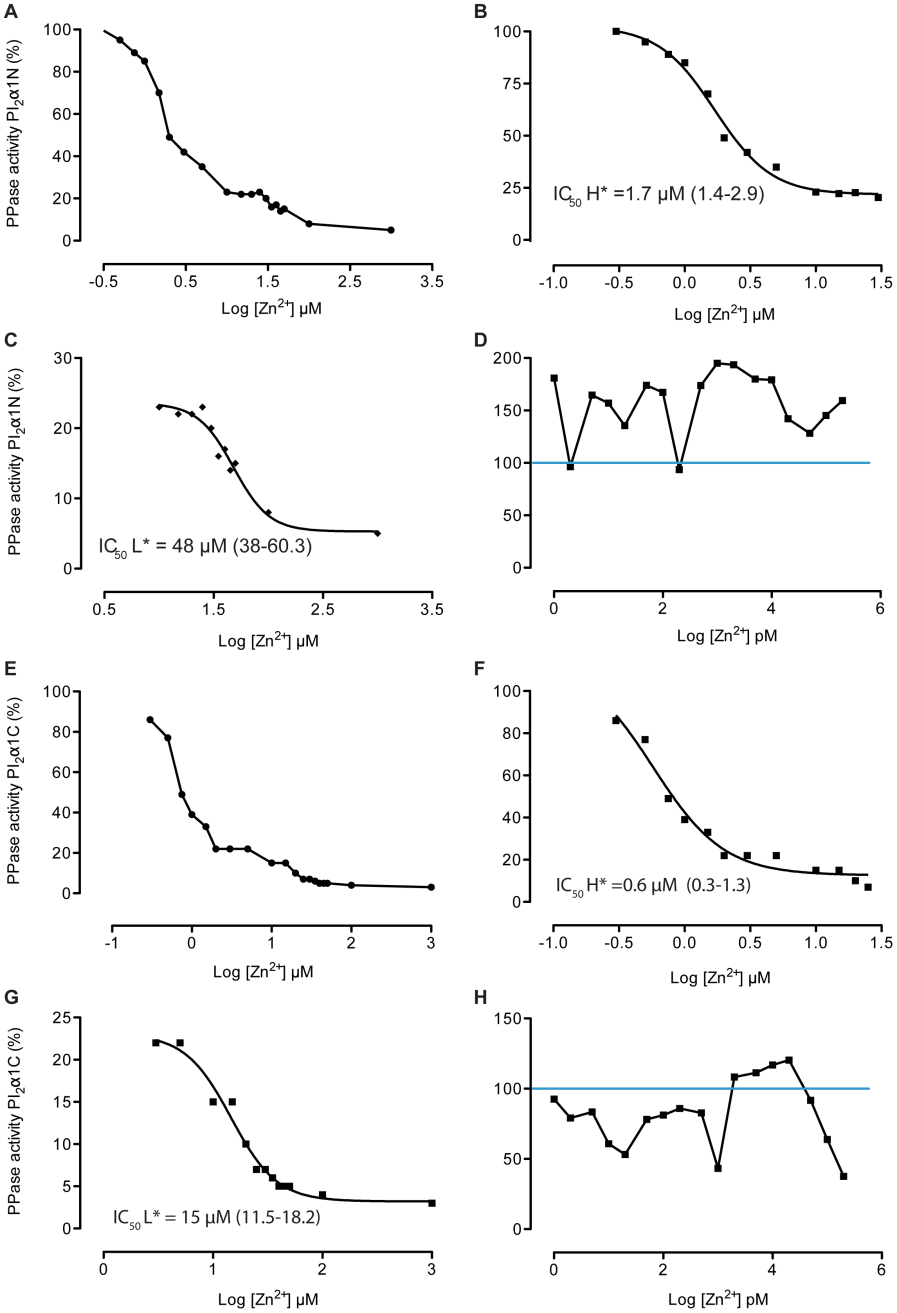
Inhibition profiles of the phosphatase (PPase) activities by Zn^2+^ ions using N-and C-terminal phosphopeptides as substrate. Assays were performed in presence of rat washed cortical membrane at 50 µg/ml of total protein concentration, with increasing amounts of Zn^2+^ ions in 10 mM Hepes buffer pH 7.4 with 1 mM Mg^2+^ in presence of. Incubations were performed at 30°C during 10 min. The activities are expressed as a percentage of that of control samples. (A) Total curve inhibition by Zn^2+^ (0.1 µM–1 mM) with pI_2_α1N-P at 10 µM as substrate, the curve could be fitted to a two-step mechanism: ‘H’ high affinity (B), and ‘L’ low affinity (C). (D) At very low Zn^2+^ concentrations (1 pM–200 nM) activation of PPase activity was observed. (E) Total curve inhibition by Zn^2+^ (0.1 µM–1 mM) in presence of pI_2_α1C-P at 10 µM, two affinities was observed: ‘H’ high affinity (F), and ‘L’ low affinity (G). (H) PPase activity at very low Zn^2+^ concentrations (1 pM–200 nM). The IC_50_ values are mentioned with their 95% confidence intervals and calculated from nonlinear regression curve fits using GraphPad Prism 5.

Previous work [22] showed that Zn^2+^ inhibited membrane-bound phosphatase activity at concentrations between 0.1 µM-1 mM. It was therefore important to confirm this observation by the present method using pI_2_α1N-P and pI_2_α1C-P as substrates (**Figure 3**). All of the phosphatases activities were completely inhibited by 1 mM Zn^2+^ using either substrate (**Figure 3A, 3E**). Two apparent IC_50_ for Zn^2+^ were determined for each peptide: high affinity (*H*) at 1.7 µM (1.4–2.9 µM confidence interval) (**Figure 3B**) and low affinity (*L*) at 48 µM (38.0–60.3 µM) (**Figure 3C**) for pI_2_α1N-P. For the C-terminal substrate pI_2_α1C-P two affinities were also observed with an IC_50_ *(H)* = 0.60 µM (0.3–1.3 µM confidence interval) (**Figure 3F**) for high affinity and an IC_50_ *(L)* = 15.0 µM (11.5–18.2 µM) for low affinity (**Figure 3G**). With low concentrations of Zn^2+^ (1 pM–200 nM), we observed several activations of the phosphatasic activities using pI_2_α1N-P (**Figure 3D**), of lower extent for the C-terminal substrate (**Figure 3H**).

#### Okadaic acid inhibition

**Figure 4:**
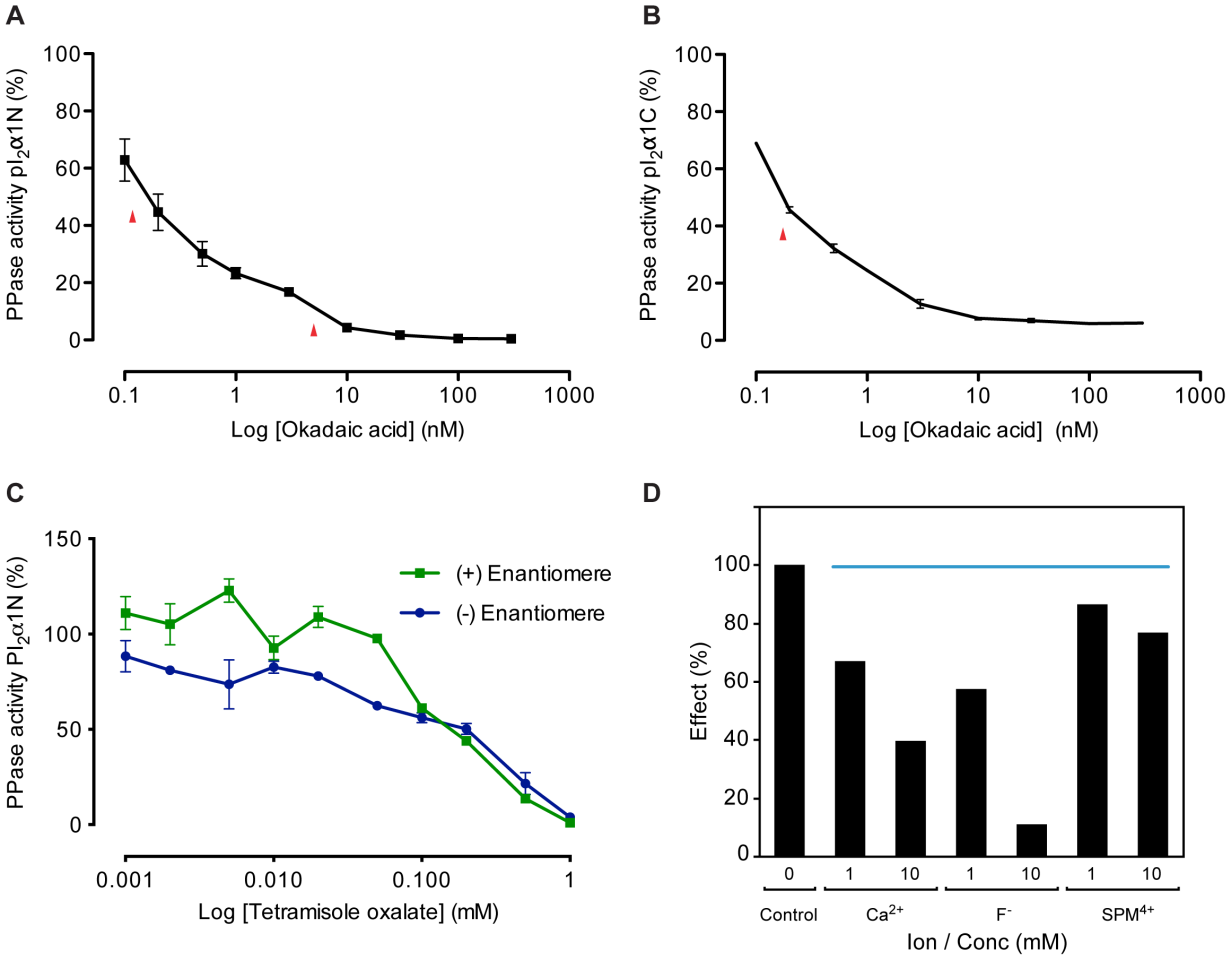
Effects of various inhibitors on the membrane-bound phosphatase activities. The phosphatase activity was performed in presence of reaction mixture contained 50 µg/ml of washed cortical membrane with 10 mM Hepes (pH 7.4) and 1 mM Mg^2+^, in addition to the inhibitor being tested. Incubations were performed at 30°C during 10 min. The activities are expressed as a percentage of that of control samples without inhibitors. Inhibition profiles of PPase activities by okadaic acid were performed using N-terminal phosphopeptide (A) or C-terminal phosphopeptide (B) as substrate. The data points are means ± SEM of 3 experiments. The red arrows indicate the different IC_50_. Using N-terminal phosphopeptide pI_2_α1N-P as substrate, membrane phosphatase activities were inhibited by both Br-t (+) and (−) enantiomers with close IC_50_ values of 0.2 and 0.1 mM respectively (C), thus excluding alkaline phosphatase. Data points are the average ± SEM of duplicate assays. (D) Effect of Ca^2+^ ions, fluoride (F^−^) and spermine (SPM^4+^) on PPase activity using N-terminal phosphopeptide as substrate.

Okadaic acid has been shown to be a potent inhibitor for PP1, PP2A, PP4 and PP5 and a weaker inhibitor for PP2B. PP2C and PP7 are not or very slightly sensitive to inhibition by okadaic acid at micromolar concentrations [29]. The differences in the IC_50_ can distinguish these phosphatases. Okadaic acid at 300 nM completely inhibits the membrane-bound phosphatases using the two phosphopeptides (**Figure 4A, B**). Two apparent IC_50_ for the inhibition of phosphatase activities by okadaic acid using pI_2_α1N-P were determined: a high affinity (*H*) at 0.15 nM and a low affinity (*L*) at 5 nM (**Figure 4A**). Only one affinity was measured using pI_2_α1C-P with an apparent IC_50_ at 0.18 nM (**Figure 4B**). These data paralleled those obtained with Zn^2+^ and confirm the presence of at least two species of membrane-bound phosphatases.

#### Inhibition of the phosphatasic activities by (+/−)-p-Br-t oxalate

The levamisole, (−) enantiomer of the *p*-bromotetramisole oxalate ((−)-*p*--Br--t) is a potent alkaline phosphatase inhibitor, whereas the same enzyme is completely insensitive to the (+) enantiomer (+)-*p*--Br--t) which is often used as a negative control [30]. We therefore tested both enantiomers with increasing concentrations (**Figure 4C**). The membrane phosphatase activities were completely inhibited by both enantiomers at 1 mM, with IC_50_ values of 0.1 mM for (+)-*p*--Br--t and 0.3 mM for (−)-*p*--Br--t, showing that the alkaline phosphatase is not involved in the dephosphorylation of the N-terminal phosphopeptide.

#### Inhibition by other ions

By the N-terminal phosphopeptide method, fluoride (F^−^) reduced phosphatase activities by 45% at 1 mM and almost 90% inhibition was observed at 10 mM, indicating an IC_50_ in the order of 2 mM. Ca^2+^ inhibited phosphatase activities at 1 mM by only 30% and by slightly less than 60% at 10 mM indicating an IC_50_ in the order of 5 mM, which is much less potent than inhibition by Zn^2+^ (**Figure 4D**).

#### Polycations inhibition

Some mammalian phosphatases are inhibited by various polycations [26]. The effects of spermine (a tetracationic polyamine) vary from one enzyme to another. Some protein tyrosine phosphatases are stimulated [31]–[33], and other phosphatases are inhibited [34] [35]. For instance, spermine inhibits both PP1 and PP2A with similar potency [36]. A more precise reason to test especially spermine among other polycations is that its content is increased in epileptogenic zones [37] where a deficiency in GABA_A_R endogenous phosphorylation is also observed [25]. It appeared therefore relevant to examine whether this polyamine has an effect on the phosphatases responsible for α1-subunit dephosphorylation. Spermine at 1 mM moderately inhibited the phosphatase activities by only (10%) and 20% at 10 mM showing that spermine is a less effective inhibitor than Zn^2+^ ions (**Figure 4D**).

### Effects of the okadaic acid on the rundown of GABA_A_ currents in acutely isolated cortical neurons

**Figure 5:**
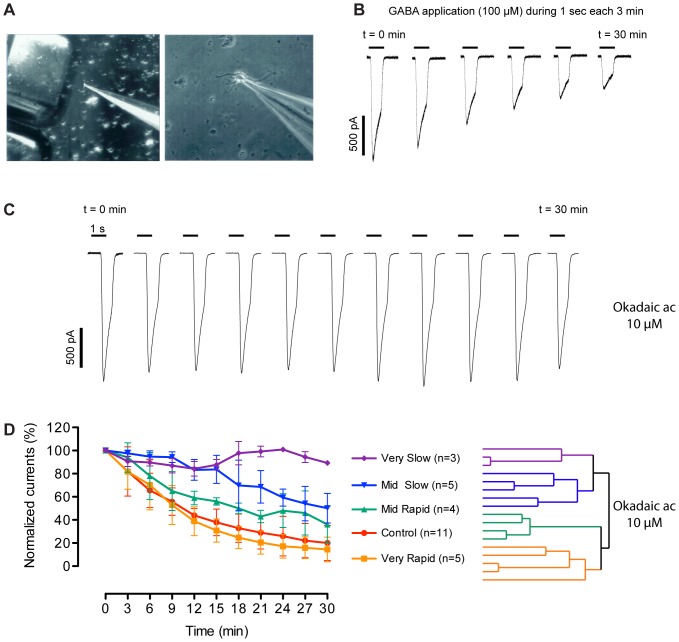
Effects of okadaic acid on the GABA_A_ current rundown. GABA_A_ currents were measured by whole-cell patch clamp on acutely dissociated cortical pyramidal neurons from Sprague Dawley rats. (A) Micrographs of a patched cell at two different magnifications, showing the rapid application device (left). (B) Rundown in cortical neuron: GABA was applied at a concentration of 100 µM during 1 second every 3 min. The maximal amplitude of GABAergic currents gradually decreased with time in control conditions. In presence of okadaic acid (10 µM in the pipette) variable effects were observed. (C) In some cells the rundown was even totally abolished. (D Left) Rundown profiles of normalized currents in presence (n = 17) or in absence (Control, n = 11) of okadaic acid; (D Right) color-coded hierarchical clustering tree for the recorded okadaic acid-treated cells in which a maximum of 4 groups are significantly distinguished: ‘Very Slow’ (n = 3), ‘Mid Slow’ (n = 5), ‘Mid Rapid’ (n = 4) and ‘Very Rapid’ (n = 5). For each group the plot (Left) is the average of normalized currents. Error bars indicate the SEM. One-way ANOVA with Dunnett’s test of mean currents and Student *t*-test were used (see in Results) indicating very likely that more than one phosphatase are involved.

We measured the rundown of the GABA-induced currents in pyramidal neurons acutely dissociated from rat cortex (**Figure 5A**). Rundown of GABA_A_ currents is a time-dependent response decrease due to a dephosphorylation process [7] (**Figure 5B**), which does not require the presence of the receptor agonist GABA, thus differing from the observed desensitization process during GABA applications. We had previously shown that the phosphatase inhibitor okadaic acid (10 µM) added to the pipette milieu has a variable effect on the GABA_A_ current rundown in four neurons [20]. As illustrated in **Figure 5C**, the rundown could be abolished in some cells. To clarify this variable effect, okadaic acid was assayed on a larger number of rat pyramidal cells (n = 17). Normalized maximal responses of neurons displayed different effects with okadaic acid (**Figure 5D**). We applied the hierarchical clusters test to the parameter of the ‘mean normalized currents’ (from t = 3 to t = 30 min) for all okadaic acid-treated neurons. Four groups of neurons were significantly distinguished by ranking rundown velocity as ‘Very Rapid’ – ‘Mid Rapid’ – ‘Mid Slow’ – ‘Very Slow’. In addition, the whole group of okadaic acid-treated cells was significantly different from the control cells group (p<0.01 **), using a two-tailed Student's *t*-test assuming unequal variances, in spite of the broad individual variation induced by addition of the phosphatase inhibitor. The 4 groups were considered separately using one-way ANOVA with Dunnett's *post hoc* test: the ‘Very Rapid’ group which was not sensitive to okadaic acid was identical to control (Ns), and the 3 other groups of neurons which responded to okadaic acid were statistically different from the control (p<0.01** for ‘Mid Rapid’, p<0.0001*** for both ‘Mid Slow’ and ‘Very Slow’). This functional approach thus also shows heterogeneity in terms of phosphatase activities in pyramidal neurons.

## Discussion

We have developed an analytical tool to characterize for the first time the membrane-bound GABA_A_-R α1-specific phosphatase activities, which were never previously identified or characterized. These phosphatases can be considered as novel targets for antiepileptic drugs research. Indeed, in recent clinical studies it was proposed to modulate the activity of protein phosphatases for therapeutic benefits [38].

Improved LC-MS-based techniques led to reliable alternatives for peptide analysis and protein quantification [39]. A known limitation in proteomic analysis of membrane proteins by mass spectrometry is the difficulty raised by their amphipathic nature [40]. Thus, detection and characterization of phosphatase activities in membrane preparations by LC-MS/MS analysis and phosphopeptides as substrate is a method not used until now, and its development was a challenge.

We used two fragments specific for the intracellular loop of α1 subunits, which can be used for all mammalians. The principle of the present phosphatase assay by LC-MS/MS is based on the measurement of relative MS signals of both phosphopeptides and native peptides resulting from dephosphorylation. We initially characterized these peptides by examining their specific MS/MS spectra of the related dominant ions. The advantage of this method was to analyze simultaneously native and phosphorylated peptides. Assaying washed brain membranes including human cortical membranes as a source of phosphatase activity we observed an important dephosphorylation by transformation of N- and C-terminal phosphopeptides into native peptides, showing thus that the membrane-bound phosphatases recognized our I_2_α1 probes as substrates. This result was crucial since it was a challenge to study such a complex membrane proteins preparation and was a prerequisite to characterize these phosphatases.

A comparative analysis of kinetics parameters of these phosphatases (**Table 1**) showed that the N-terminal substrate was more efficient (higher specific activity and higher affinity) than the C-terminal one. For the N-terminal phosphopeptide the Lineweaver-Burk plot was not linear. This point was further studied using slope tests: two significantly different apparent affinities were observed. We noted a reduction of the phosphatase activity heterogeneity of C-terminal comparing to the N-terminal substrate. This was confirmed by the data obtained by okadaic acid inhibition, where we observed two distinct affinities for N-terminal substrate and only one apparent affinity for C-terminal. It has been shown that some phosphatases have indeed a better substrate preference for phosphothreonine than for phosphoserine residues in short peptides [41]. These observations strongly support the hypothesis of several phosphatases regulating the GABA_A_R function.

The protein phosphatases are generally classified according to their substrate specificity, ion requirement and sensitivity to inhibitors, and are divided into two major categories-protein: serine/threonine phosphatases (PSTPs) [42] and tyrosine phosphatases (PTPs) which include dual-specificity phosphatases (DUSPs) [43]. Two distinct PSTPs gene families have been described: PPM and PPP. The protein Ser/Thr phosphatases PP1, PP2A, and PP2B of the PPP family, together with PP2C of the PPM family, account for the majority of the protein serine/threonine phosphatase activity. The PPM family is composed of Mg^2+^-dependent phosphatase including PP2C, pyruvate dehydrogenase phosphatase, and PP2C-“like” phosphatases [44]. These enzymes play a key role in neuronal plasticity, learning and memory [16], and in several neurological disorders such as amyotrophic lateral sclerosis and epilepsy [45]. An important issue is to identify the metal ions required for optimal phosphatase activity. Both PP1 and PP2A are active in the absence of divalent cations, whereas PP2B and PP2C have respectively an absolute requirement for Ca^+2^ and Mg^2+^ [36].

The activity of membrane-bound phosphatases was tested in the presence of a variety of factors that can interfere with these enzymes. Addition of increasing concentrations of Mg^2+^ exhibited moderate effects on activation of phosphatase activities, and in the presence of chelators a residual phosphatase activity was observed, revealing the presence of both Mg^2+^-dependent and Mg^2+^-independent protein phosphatases, indicating again the heterogeneity of these enzymes. A similar heterogeneity was reported for the atrial natriuretic peptide receptor (NPR-A) phosphorylated on 4 serine and 2 threonine residues by an unknown kinase(s) and dephosphorylated by two unknown phosphatases, the activity of which was dependent or not on Mg^2+^ [46].

Ca^2+^, fluoride and spermine at millimolar concentrations inhibited the phosphatase activities. The weak inhibition by spermine was interesting since its content is increased in the epileptogenic tissue [37]. One may speculate that such inhibition could counteract the increased GABA_A_ current rundown observed in neurons isolated from human epileptogenic cortical tissue. However, the very high IC_50_ (>10 mM) of spermine inhibition makes such compensation unlikely, since millimolar spermine concentrations were never observed in the human cortical tissue, even in pathological conditions.

Inhibition observed with *p*-bromotetramisole oxalate (*p*-Br-t) allowed us to discard the alkaline phosphatase as an involved enzyme in the present mechanism. It has been shown that *p*-Br-t can inhibit other phosphatases such as PP1, PP2A, PP2B and PP2C with variable efficacy [30]. The same authors showed an inhibition by both enantiomers for PP2A and PP2C, however with different IC_50_ in the millimolar range. They also observed that the (+) enantiomer is more potent than the (−) enantiomer for PP2C. Likewise, we showed that inhibition by (+)-*p*-Br-t was slightly more effective than that by (−)-*p*-Br-t for the membrane-bound phosphatase activity using the N-terminal phosphopeptide. However, the IC_50_ we measured were 3–7 fold lower than those for PP2C and PP2A. Thus these classical phosphatases were very unlikely involved in the GABAergic α1 mechanism.

We had previously showed that sodium orthovanadate inhibits membrane phosphatases [22] and reduced rundown of GABA_A_ currents [20]. This phosphate analog could not be used in our conditions for phosphatase characterization, because a complete ESI-MS/MS signal extinction occurred in positive mode occurred, likely due to the negative charge and non-volatility of the VO_4_^3−^ anion. Indeed, all ESI-MS/MS manufacturers totally prohibit the use of phosphate buffers for the same reason [47].

It was of special interest to investigate the zinc-sensitivity of the membrane-bound phosphatase, since zinc oxide was known to present anti-epileptic effects and used for this purpose during the 19^th^ century [48]. The effect of Zn^2+^ is variable depending on the phosphatases: these ions as well as Fe^2+^ are activators at the catalytic site of PP1, considered as a metallo-enzyme [49], whereas Zn^2+^ inhibits the PP2C class by competition with Mg^2+^/Mn^2+^ at the metal binding site of the catalytic domain [26], [28]. We had previously shown in washed brain membranes that complete inhibition of GABA_A_R dephosphorylation occurred with Zn^2+^ using the autoradiographic method [22]. In the present study we found that Zn^2+^ in the micro- to millimolar ranges was one of the most potent tested phosphatase inhibitors. Two apparent affinities were determined using both N- and C-terminal phosphopeptides. Free cellular Zn^2+^ concentrations (picomolar range) are orders of magnitudes lower than the total cellular Zn^2+^ content (nanomolar range) [50]. In these ranges, we observed a series of activations of phosphatasic activities for the N-terminal substrate. Our washed cortical membrane preparations are cytosol free, without proteins chelating Zn^2+^ such as metallothioneins [51]. Therefore, the observed multiple activations of phosphatasic activities by zinc are rather reflecting different affinities of distinct phosphatases for these ions. These data indicate again the heterogeneity of the membrane-bound phosphatases dephosphorylating GABA_A_R α1 subunits. Unfortunately, the effect of Zn^2+^ could not be tested directly on GABA_A_ receptor currents in whole-cell patch-clamp of acutely isolated pyramidal neurons because Zn^2+^ addition into the pipette milieu systematically induced breakdown of the nano-seal at the tip of the glass micropipettes (data not shown).

The organic inhibitors such as okadaic acid are very useful to distinguish some classes of protein phosphatases [52]. Okadaic acid inhibits various phosphatases in mammalian brain with very distinct IC_50_ values: PP1 (270 nM), PP2A (2 nM), PP2B (3.6 µM), PP4 (0.2 nM), PP5 (1.4–10 nM) and PP6 (2 nM) whereas PP2C, PTP, PP7 acid phosphatase and alkaline phosphatase were not inhibited by up to 1–10 µM okadaic acid [36]. We showed that it strongly inhibited phosphatase activities but the apparent IC_50_ values were different from those described for the classical phosphatases, suggesting that the membrane-bound phosphatases are not yet identified.

The rundown of the GABA_A_R-mediated responses in dissociated neurons involves an endogenous phosphorylation–dephosphorylation process [7], [20]. Okadaic acid at 10 µM was tested on the GABA_A_ response rundown. Four groups of cortical neurons were distinguished suggesting that each group could correspond to distinct phosphatase types. The group showing no inhibition indicates the presence in these cells of okadaic-resistant phosphatase activity. The 3 groups with different sensitivities to this inhibitor suggest the presence of at least two distinct classes of phosphatases, a further argument for their heterogeneity. In addition, okadaic acid at 10 µM inhibited most phosphatasic activities by the phosphopeptide method. This observation suggests that in the course of preparation of the washed cortical membranes, a loss of some GABA_A_R α1 subunit phosphatases weakly attached at the membranes occurred.

We therefore clearly propose heterogeneity of the phosphatases regulating GABA_A_R endogenous phosphorylation at the cellular level. In addition to the above-mentioned case of NPR-A, other examples of dephosphorylation of a single protein by multiple distinct Ser/Thr phosphatases are known, such as centrin [53] and [54]. The α1-subunit dephosphorylation by multiple phosphatases in GABA_A_R likely reflects alternate modulations of the inhibitory neurotransmission, and also different functional responses to ions and endogenous effectors in neurons. Since the involved phosphatases do not correspond to the classical enzymes, it will be of great interest to isolate these membrane-bound phosphatases and to identify them in a future study.

### Conclusion and prospects

We have developed an analytical tool using LC-MS/MS method to study membrane-bound phosphatases specific to α1 GABA_A_R, including those prepared from human brain tissue. This method is highly sensitive and can be applied to crude extracts as well as to purified enzymes and would be useful for membrane phosphatases assays. Our main finding is the heterogeneity of the phosphatases dephosphorylating the GABA_A_R **α**1 subunit. Atypical pharmacological profiles were obtained for these phosphatases as shown by the zinc-sensitivity, excluding classical phosphatases even though sharing some of their properties. A challenge for future studies will be to purify these different membrane-bound phosphatases using the present LC-MS/MS method for monitoring. Identifying the membrane-bound phosphatases of GABA_A_ receptors will improve our understanding of the molecular mechanisms modulating GABAergic function and their alterations in patients with drug-resistant seizures. These advances would also provide the basis for development of new antiepileptic drugs by targeting the phosphatases specific to the GABA_A_R α1 subunit.

## Supporting Information

Figure S1 **Quantification standard curves of peptides under incubation conditions of phosphatasic activity.** Native (A) and phosphorylated (B) N-terminal peptides, (C) native and (D) phosphorylated C-terminal peptides were prepared at different concentrations in 10 mM Hepes buffer, 1 mM Mg^2+^ and acetic acid 10% (w/v). Regression lines present chromatogram peak area of MS/MS spectra depending on peptides concentrations. Determination coefficient (r^2^) was calculated using GraphPad Prism 5.
(TIF)

Figure S2 **Quantification standard curves of N-terminal peptides in biological matrix using washed cortical membranes.** Native (A) and phosphorylated (B) N-terminal peptides were prepared at different concentrations in presence of 50 µg/ml (total proteins) of washed cortical membrane in 10 mM Hepes-Tris buffer pH 7.4 with 1 mM MgCl_2_ incubated at 30°C for 10 min, the same conditions as for phosphatasic assays. For phosphopeptide, acetic acid 10% (w/v) was added before the membrane preparation in order to prevent dephosphorylation.
(TIF)

Figure S3 **Double quantification of N-terminal phospho- and native peptide concentrations after dephosphorylation.** Phosphatasic reactions were performed in presence of N-terminal phosphopeptide (10 µM) at different protein concentrations of washed cortical membranes (1-3-10-30-100 and 300 µg/ml). The total amount (by 2-µl sample) of phosphopeptide and of native peptide was constant, with an average of 20.43 pmol (SEM = 0.32, n = 6).
(TIF)
