# Supplementary figures and images for: Mass Spectrometric Detection and Characterization of Atypical Membrane-Bound Zinc-Sensitive Phosphatases Modulating GABAA Receptors
Source: PLoS One. 2014 Jun 26;9(6):e100612. doi: 10.1371/journal.pone.0100612 (PMC4072668; doi:10.1371/journal.pone.0100612)

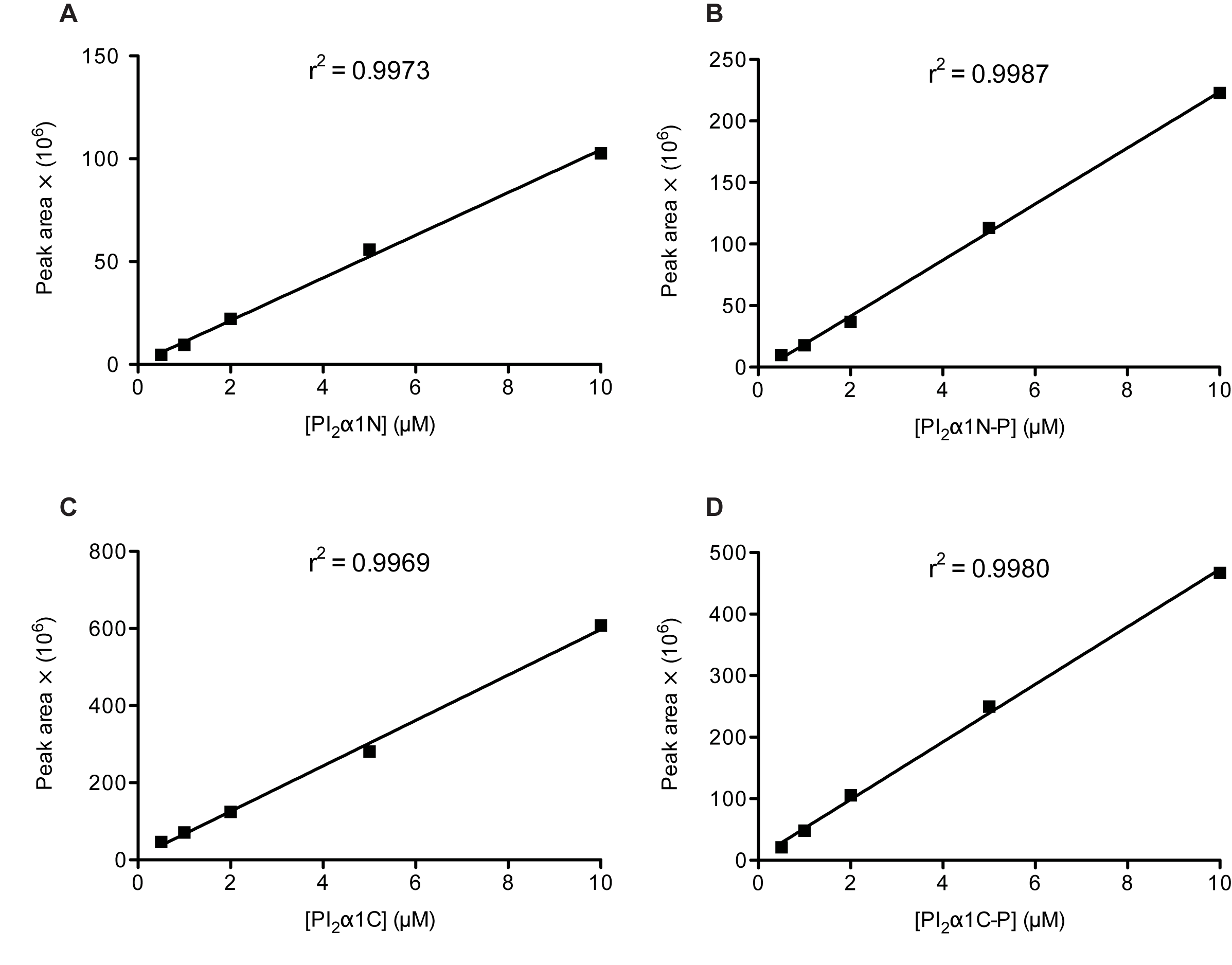

Supplement: Figure S1 — Quantification standard curves of peptides under incubation conditions of phosphatasic activity. Native (A) and phosphorylated (B) N-terminal peptides, (C) native and (D) phosphorylated C-terminal peptides were prepared at different concentrations in 10 mM Hepes buffer, 1 mM Mg2+ and acetic acid 10% (w/v). Regression lines present chromatogram peak area of MS/MS spectra depending on peptides concentrations. Determination coefficient (r2) was calculated using GraphPad Prism 5. (TIF) [file pone.0100612.s001.tif]

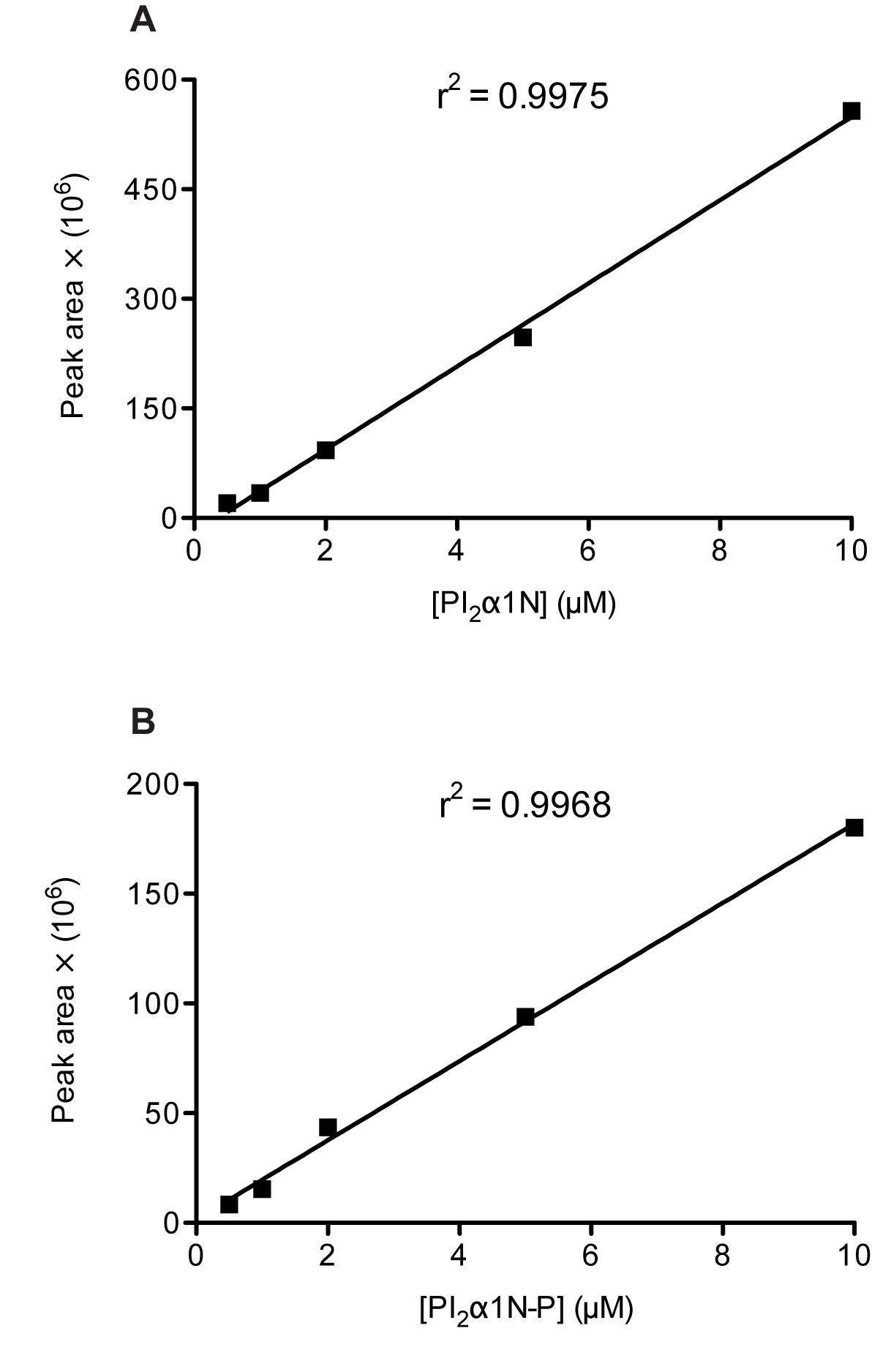

Supplement: Figure S2 — Quantification standard curves of N-terminal peptides in biological matrix using washed cortical membranes. Native (A) and phosphorylated (B) N-terminal peptides were prepared at different concentrations in presence of 50 µg/ml (total proteins) of washed cortical membrane in 10 mM Hepes-Tris buffer pH 7.4 with 1 mM MgCl2 incubated at 30°C for 10 min, the same conditions as for phosphatasic assays. For phosphopeptide, acetic acid 10% (w/v) was added before the membrane preparation in order to prevent dephosphorylation. (TIF) [file pone.0100612.s002.tif]

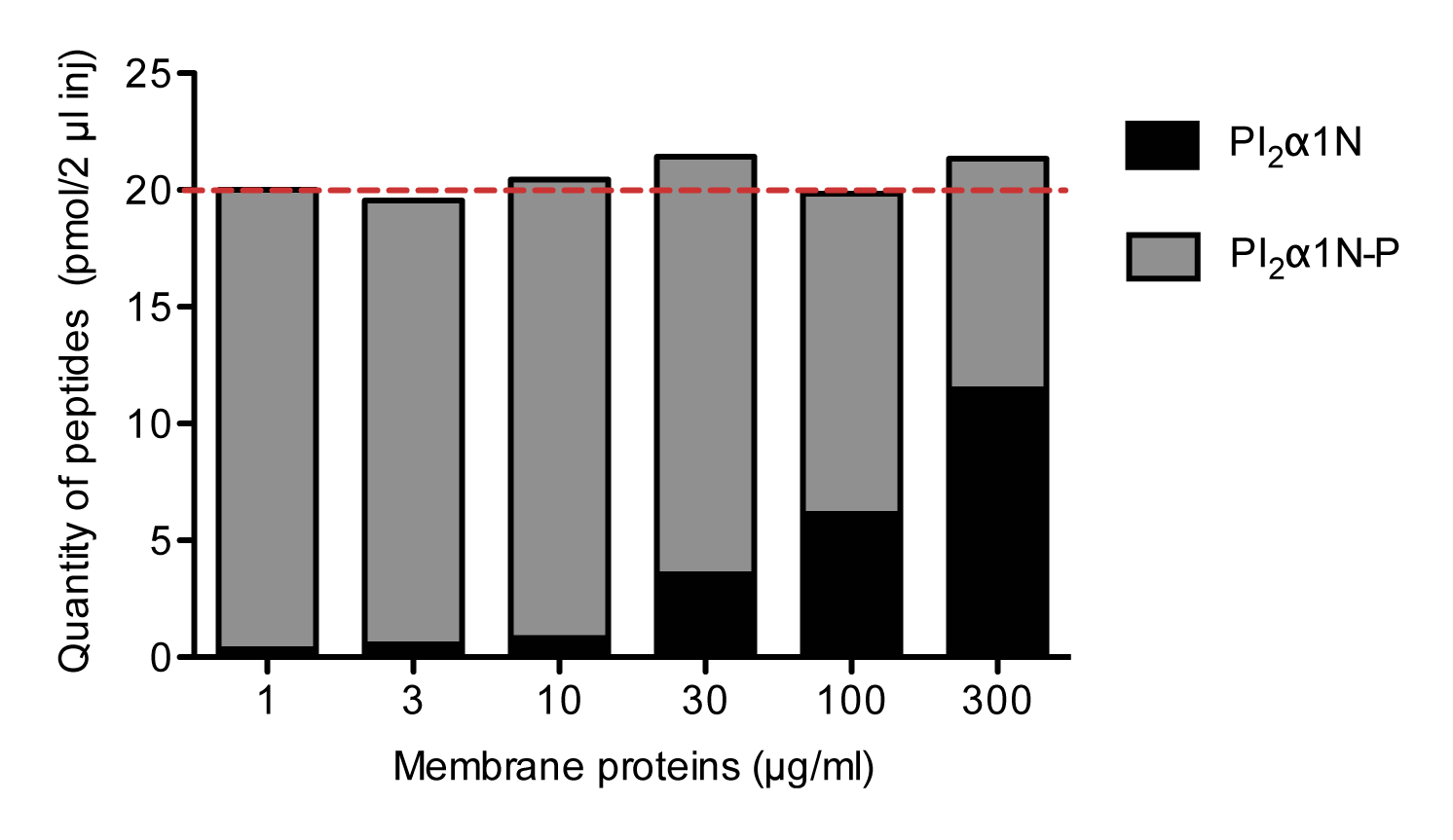

Supplement: Figure S3 — Double quantification of N-terminal phospho- and native peptide concentrations after dephosphorylation. Phosphatasic reactions were performed in presence of N-terminal phosphopeptide (10 µM) at different protein concentrations of washed cortical membranes (1-3-10-30-100 and 300 µg/ml). The total amount (by 2-µl sample) of phosphopeptide and of native peptide was constant, with an average of 20.43 pmol (SEM = 0.32, n = 6). (TIF) [file pone.0100612.s003.tif]
